# Supplementary material for: ZNF524 directly interacts with telomeric DNA and supports telomere integrity
Source: Nat Commun. 2023 Dec 12;14:8252. doi: 10.1038/s41467-023-43397-7 (PMC10716145; doi:10.1038/s41467-023-43397-7)
Supplement: Supplementary file 1 — Supplementary Information [file 41467_2023_43397_MOESM1_ESM.pdf]

# Supplementary Information for

## ZNF524 directly interacts with telomeric DNA and supports telomere integrity

Hanna Braun, Ziyan Xu, Fiona Chang, Nikenza Viceconte, Grishma Rane, Michal Levin, Liudmyla Lototska, Franziska Roth, Alexia Hillairet, Albert Fradera-Sola, Vartika Khanchandani, Zi Wayne Sin, Wai Khang Yong, Oliver Dreesen, Yang Yang, Yunyu Shi, Fudong Li*, Falk Butter*, Dennis Kappei*

* Correspondence should be addressed to Dennis Kappei (dennis.kappei@nus.edu.sg), Falk Butter (f.butter@imb.de) or Fudong Li (lifudong@ustc.edu.cn)

**This file includes:**

Supplementary Figures 1 to 8

Supplementary Tables 1 to 3

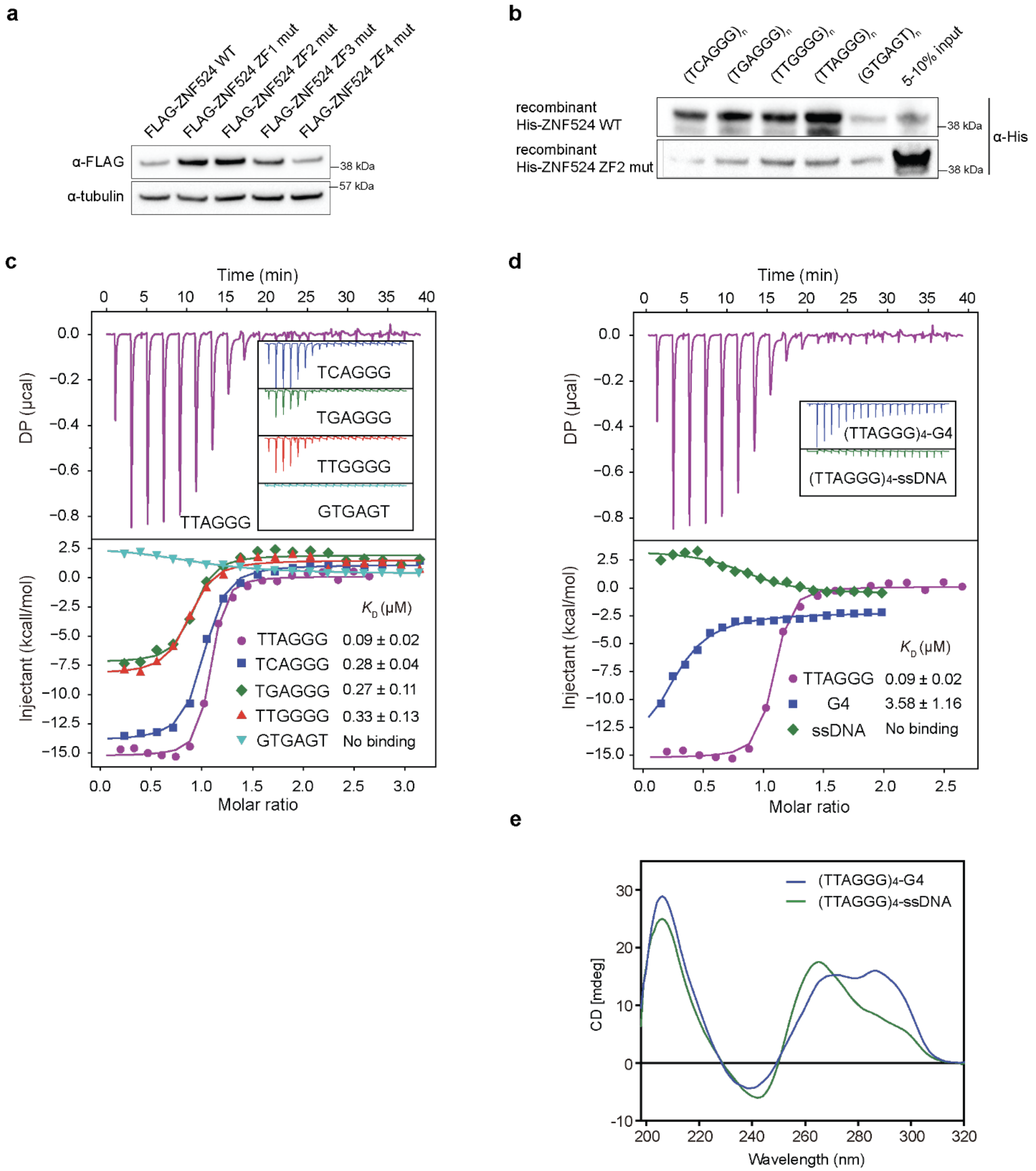


**Supplementary Fig. 1. ZNF524 directly binds TTAGGG repeats and variant repeats**

(**a**) Protein abundance of ZF mutants transiently expressed in HEK293. Tubulin served as loading control (n=2 with each replicate seeded and transfected independently). (**b**) DNA pulldowns with canonical and variant telomere repeats with bacterially expressed His-ZNF524 WT and His-ZNF524 ZF2 mutant (n=2 with each replicate expressed independently). (**c**) Isothermal titration

calorimetry results using a ZNF524 minimal domain containing only the four zinc fingers (110-223 aa) with the telomeric 12-bp ds(TTAGGG)$_2$ (same data as shown in Fig. 1d) and the telomeric variants ds(TCAGGG)$_2$, ds(TGAGGG)$_2$ or ds(TTGGGG)$_2$; ds(GTGAGT)$_2$ serves as negative control sequence; $K_D$ values with standard deviations are noted in the lower right corner. (**d**) Isothermal titration calorimetry results using a ZNF524 minimal domain containing only the four zinc fingers (110-223 aa) with the telomeric 12-bp ds(TTAGGG)$_2$ (same data as shown in Fig. 1d), the telomeric repeats forming a G4 [(TTAGGG)$_4$ – G4] or telomeric ssDNA [(TTAGGG)$_4$ – ssDNA]; $K_D$ values with standard deviations are noted in the lower right corner. (**e**) CD spectra of (TTAGGG)$_4$ forming G4 in 100 mM KCl buffer or remaining ssDNA in 100 mM NaCl buffer.

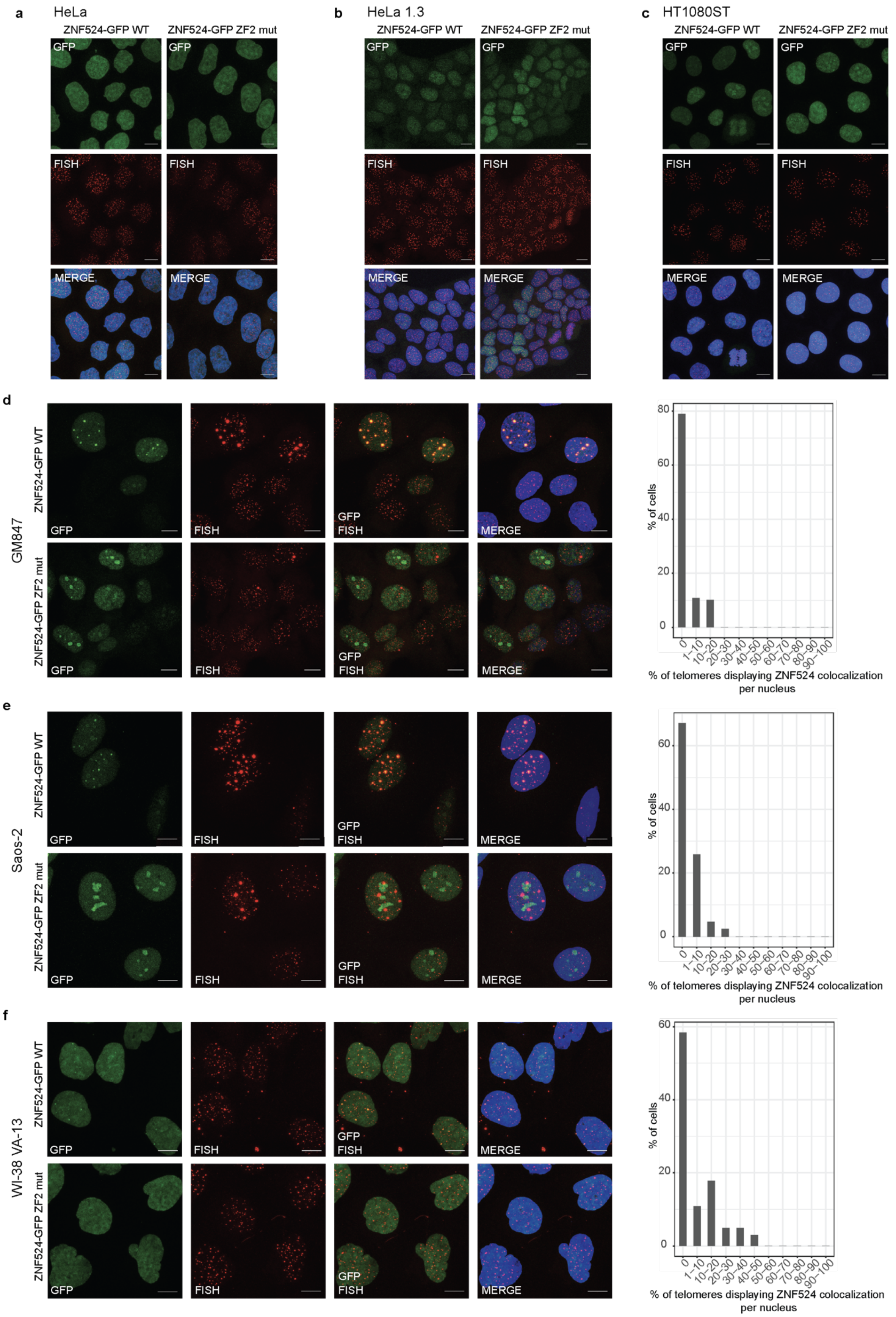

a
HeLa
ZNF524-GFP WT
ZNF524-GFP ZF2 mut
GFP
FISH
MERGE
b
HeLa 1.3
c
HT1080ST
d
GM847
e
Saos-2
f
WI-38 VA-13
% of cells
% of telomeres displaying ZNF524 colocalization per nucleus
0
1-10
10-20
20-30
30-40
40-50
50-60
60-70
70-80
80-90
90-100

**Supplementary Fig. 2. ZNF524 localizes to telomeres in ALT cells**

Fluorescence microscopy of colocalization between telomeric FISH (red) and GFP-ZNF524 (green). Representative images of doxycycline induced ZNF524-GFP WT and the ZF2 mutant in HeLa (**a**), HeLa1.3 (**b**), HT1080ST (**c**), GM847 (**d**), Saos-2 (**e**), and WI-38 VA-13 (**f**) cells are shown (scale bar 10 µm). Nuclei were counterstained with DAPI (blue). Quantification of colocalization events of ZNF524-GFP WT with telomeric PNA in GM847 (**d**), Saos-2 (**e**), and WI-38 VA-13 (**f**) (n=148, 86 or 101 nuclei respectively). Telomeric foci and the overlap with GFP foci were scored on maximum intensity projections of the acquired z-stacks.

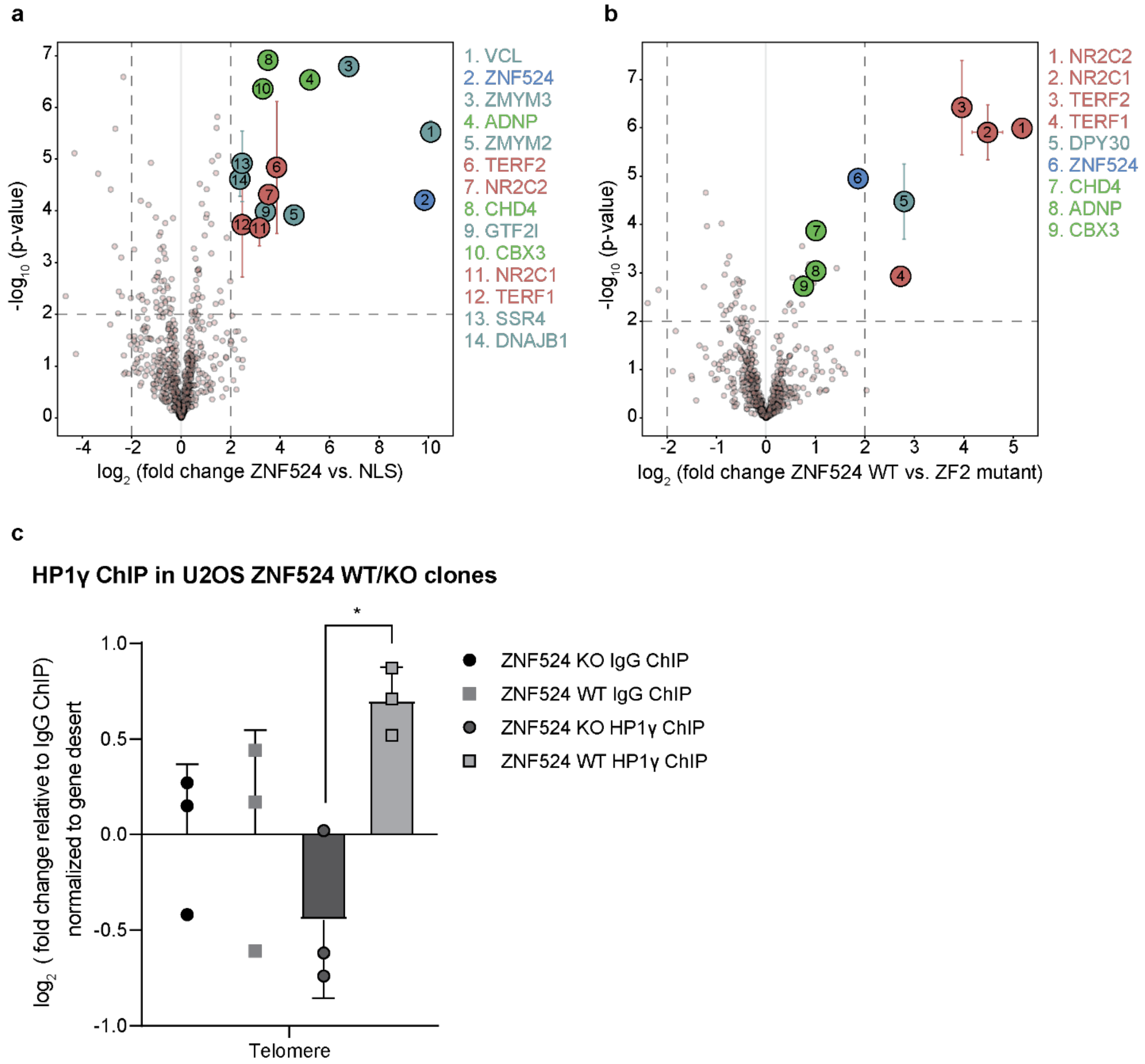


**Supplementary Fig. 3. ZNF524 associates with the ChAHP complex and affects telomeric HP1γ abundance**

(**a**) Volcano plot of BioID assay comparing proximity partners of ZNF524 WT versus NLS in U2OS cells. BirA*-ZNF524 WT and BirA*-NLS were induced with 300 and 40 ng ml$^{-1}$ doxycycline, respectively. Specifically-enriched proteins (colour-filled numbered circles) are distinguished from background binders by a >4-fold enrichment and p<0.01 (two-sided Student's t test, n=4 with each replicate seeded and induced independently). Red indicates telomeric proteins, green indicates members of the ChAHP complex and blue marks ZNF524. Other enriched proteins, including epigenetic factors, are colored in teal. Two-dimensional error bars represent the standard

deviation after iterative imputation cycles during the label-free analysis with substituted zero values (e.g. no detection in the NLS reaction).

(**b**) Volcano plot as seen in Fig. 3f, additionally indicating the members of the ChAHP complex in green. (**c**) Quantification of ChIP-qPCR experiments comparing HP1γ (CBX3) abundance at telomeres in U2OS WT and ZNF524 KO cells. IgG served as negative control (n=3 with n representing the number of independent clones used as biological replicates; error bars represent SD; * $p<0.05$, one-way ANOVA).

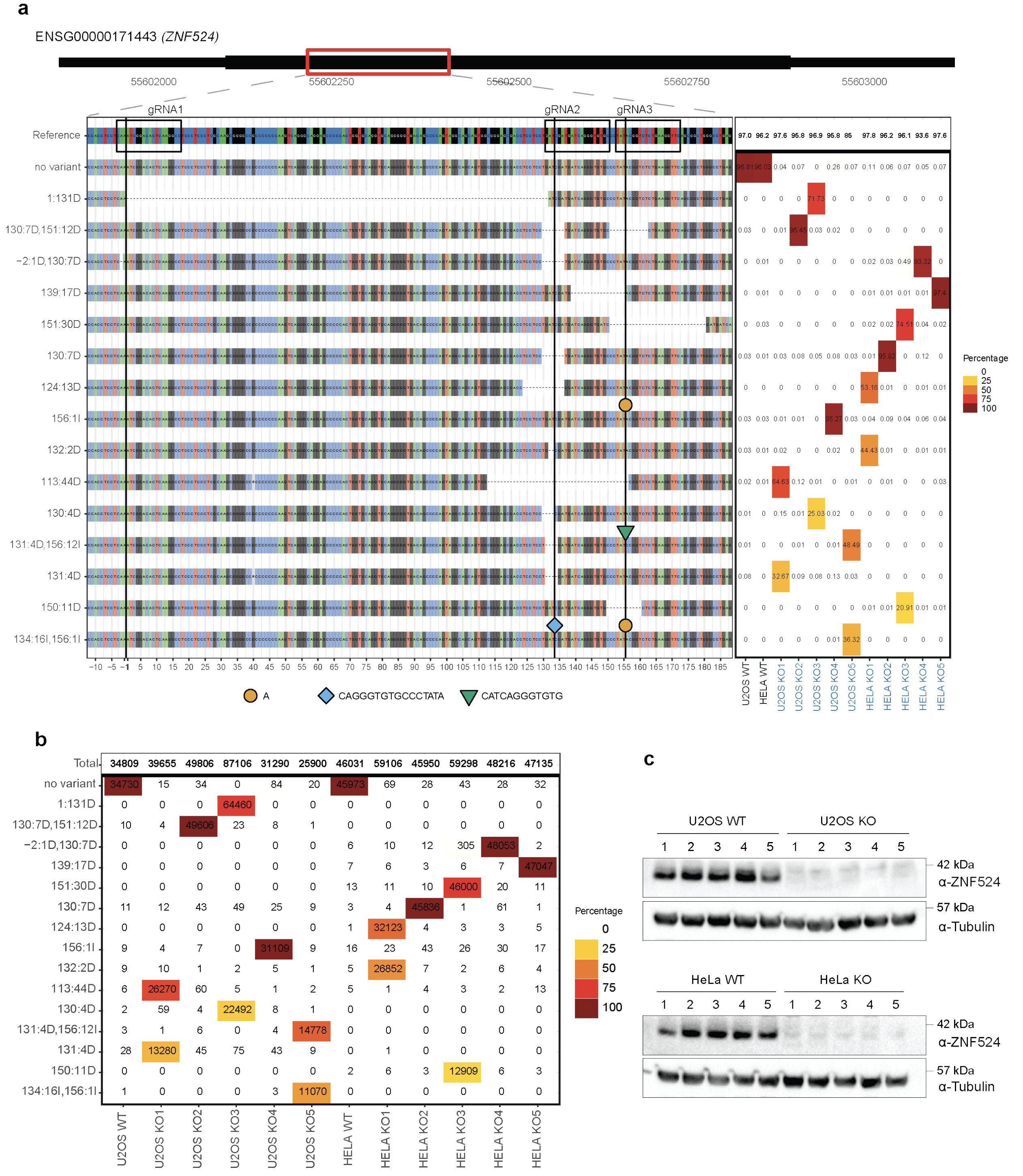


**Supplementary Fig. 4. Validation of U2OS and HeLa ZNF524 KO cell lines**

(**a**) Analysis of genomic modifications in ZNF524 KO clones by next generation sequencing. The red box indicates the position of modifications within the gene. The sequences of the different variants are plotted in comparison to the reference sequences (A: green, C: blue, G: gray, T: red).

Dotted lines indicate deletions, insertions are depicted by shape- and color-coded symbols with the exact sequences listed below the plot. The calculated allele frequencies (in %) of each KO clone are shown in the table on the right. (**b**) Absolute counts obtained for each clone by next generation sequencing. In addition, the percentage of each of these modifications per clone is color coded. (**c**) Western blots of U2OS and HeLa WT and ZNF524 KO clones with our self-produced α-ZNF524 antibody. Tubulin served as loading control (n=3 with lysates collected at different time points).

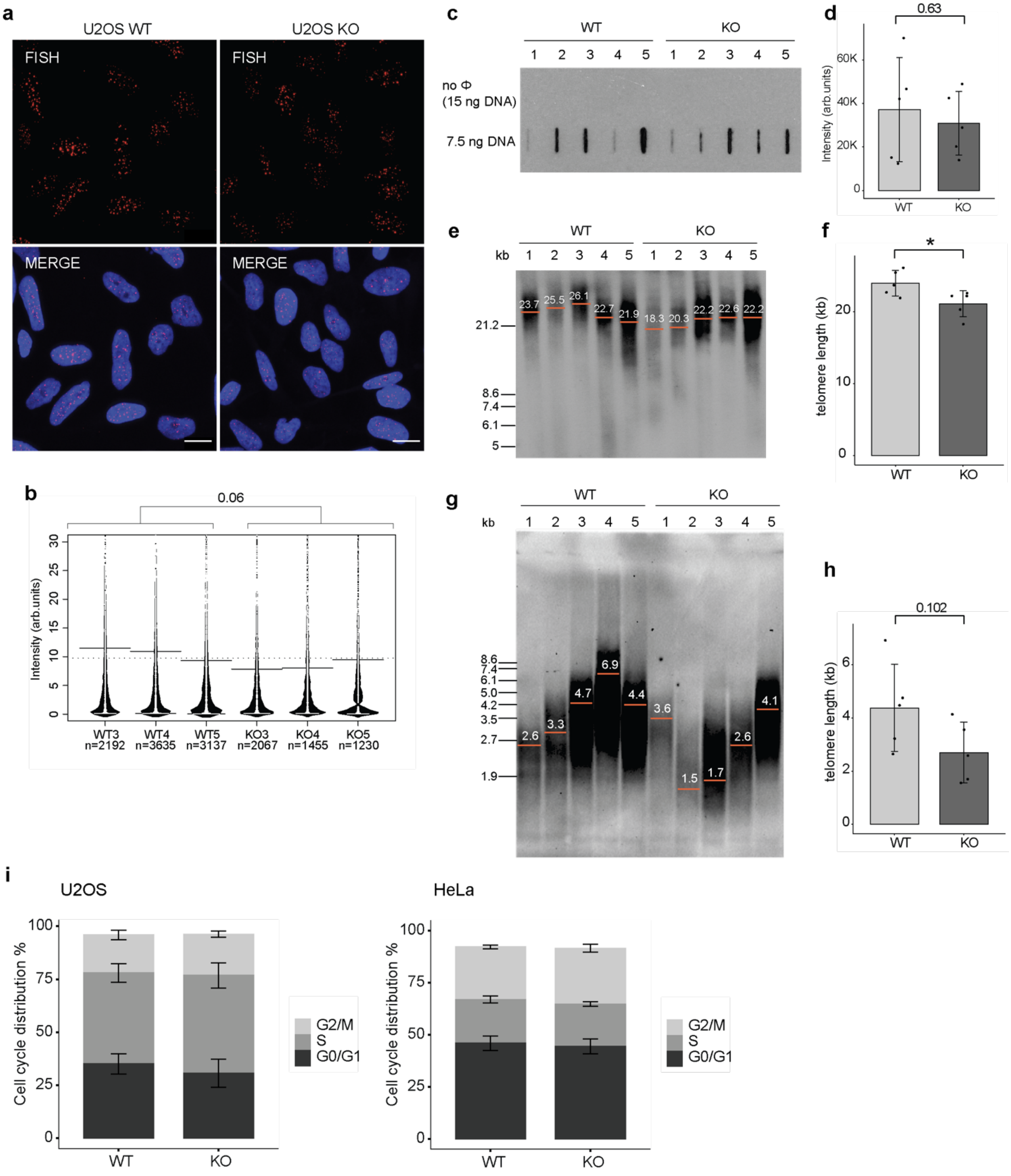

a
U2OS WT
U2OS KO
FISH
MERGE
b
0.06
Intensity (arb.units)
WT3 n=2192
WT4 n=3635
WT5 n=3137
KO3 n=2067
KO4 n=1455
KO5 n=1230
c
WT
KO
no Φ (15 ng DNA)
7.5 ng DNA
d
0.63
Intensity (arb.units)
WT
KO
e
kb
f
*
telomere length (kb)
g
h
0.102
i
U2OS
HeLa
Cell cycle distribution %
G2/M
S
G0/G1

**Supplementary Fig. 5. Effect of ZNF524 on telomere length maintenance and cell cycle progression**

(**a**) Representative images of FISH staining in U2OS WT and ZNF524 KO clones. The TAMRA-labeled C-rich telomere probe (red) was counterstained with DAPI (blue). Scale bars represent 10 µm. (**b**) qFISH analysis of the TAMRA-labeled C-rich telomere probe. The bean plot shows the individual data points as densities with the solid line representing the mean. The experiment was performed with 3 WT and 3 KO clones. n is the number of quantified foci. Student's t-test compares the mean values of each clone; the p-value is indicated above the plot (n=3). (**c**) C-circle assay of U2OS WT and ZNF524 KO clones. The slot blot shows the C-circle amplification products of 5 WT and 5 KO clones with 7.5 ng DNA template and the no φ29 polymerase negative control. (**d**) Quantification of the C-circle assay. The intensity values of the individual clones are depicted as black dots. The bar plot shows the mean intensities ± SD. Statistical significance was determined by Welch-test and the p-value stated above the graph (n=5). (**e**) TRF assay of U2OS WT and ZNF524 KO clones. The red bars and numbers indicate the average telomere length of the respective clone. (**f**) Quantification of the TRF assay of U2OS cells. The bar plot shows the mean intensities ± SD. Statistical significance was determined by Welch-test and the p-value stated above the graph (n=5). (**g**) TRF assay of HeLa WT and ZNF524 KO clones. The red bars and numbers indicate the average telomere length of the respective clone. (**h**) Quantification of the TRF assay of HeLa cells. The bar plot shows the mean intensities ± SD. Statistical significance was determined by Welch-test; *p <0.05 (n=5). (**i**) Stacked bar plot showing the cell cycle distribution of WT and ZNF524 KO cells (U2OS and HeLa) as determined by flow cytometry with PI staining. The bar plot shows the mean ± SD of 5 WT and 5 KO clones (n=5). For all data, n represents the number of independent clones used as biological replicates.

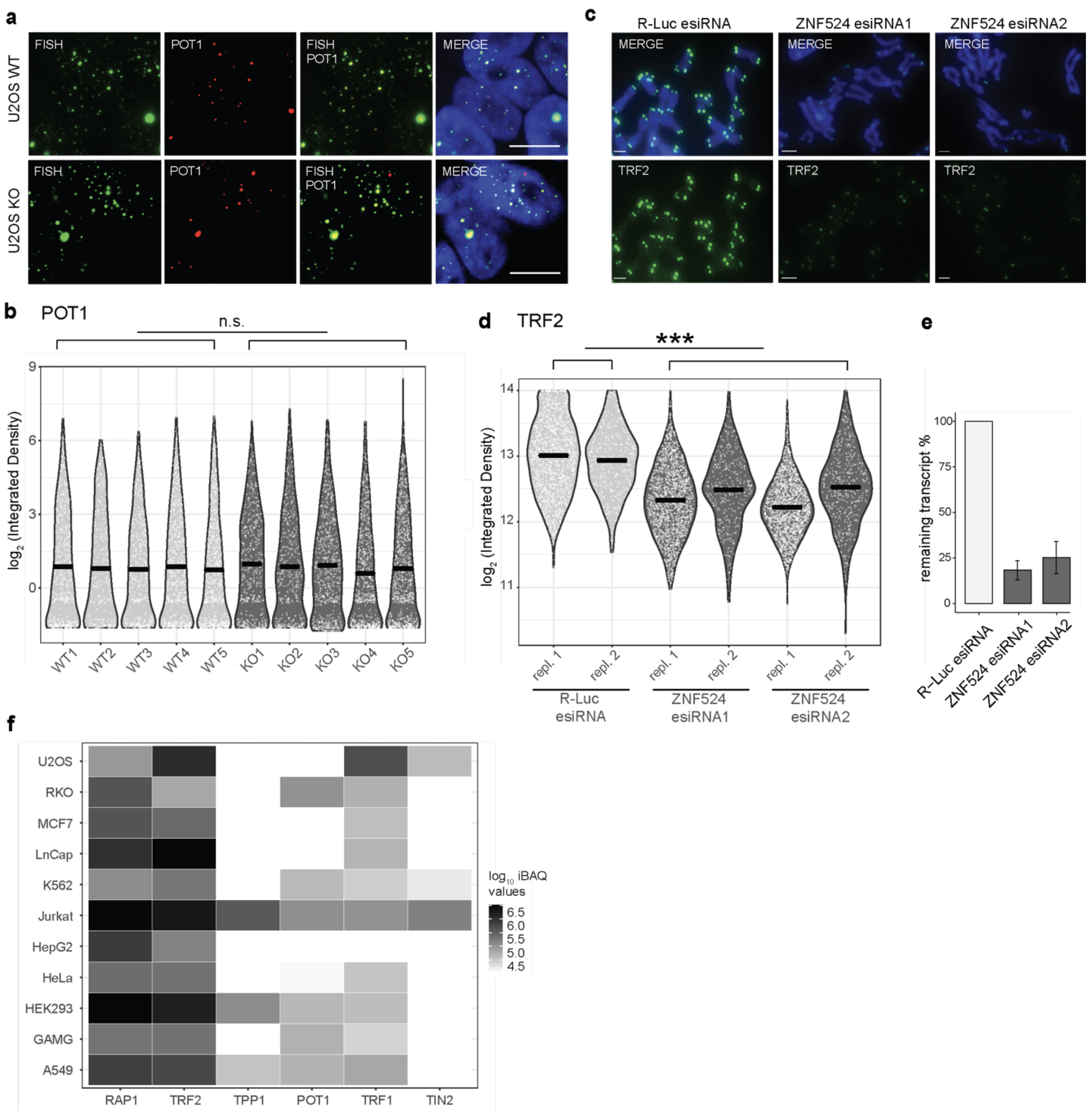


**Supplementary Fig. 6. TRF2 reduction at telomeres results from ZNF524 KO**

(**a**) Representative images of IF staining for POT1 (red) coupled to FISH staining (green) for telomeres in U2OS WT and ZNF524 KO cells. (**b**) Quantification of POT1 IF signal. The violin plot depicts the intensity values of 5 WT and 5 KO clones. 329-790 telomeres per clone were analyzed. The mean is indicated by a solid line and significance determined by Student's t-test (n=5 with n representing the number of independent clones used as biological replicates). (**c**) Representative pictures of IF staining for TRF2 (green) on metaphase spreads. U2OS cells were

treated with two different esiRNAs (1 and 2) or an esiRNA targeting Renilla luciferase (R-Luc) as negative control. Metaphases were counterstained with DAPI (blue). (**d**) Quantification of TRF2 IF signals of control (R-Luc) and ZNF524 knock-down (esiRNA1, esiRNA2) metaphases. The violin plot depicts the intensity values of the two different esiRNAs in two replicates. 1536-1981 telomeres per condition were analyzed for replicate 1 and 384-429 telomeres per condition were analyzed for replicate 2. The mean is depicted as a solid line. ***$p<0.001$, Student's t-test (n=2 with each replicate seeded and transfected independently). (**e**) Validation of ZNF524 knock-down efficiency determined by qPCR. R-Luc served as negative control (n=3-4 with each replicate seeded and transfected independently; each biological replicate represents the average of three technical replicates in the qPCR reaction). (**f**) Heatmap of shelterin member expression levels in 11 different cells lines as published in an independent study where iBAQ intensities were measured by quantitative mass spectrometry and $\log_{10}$ transformed (*35*).

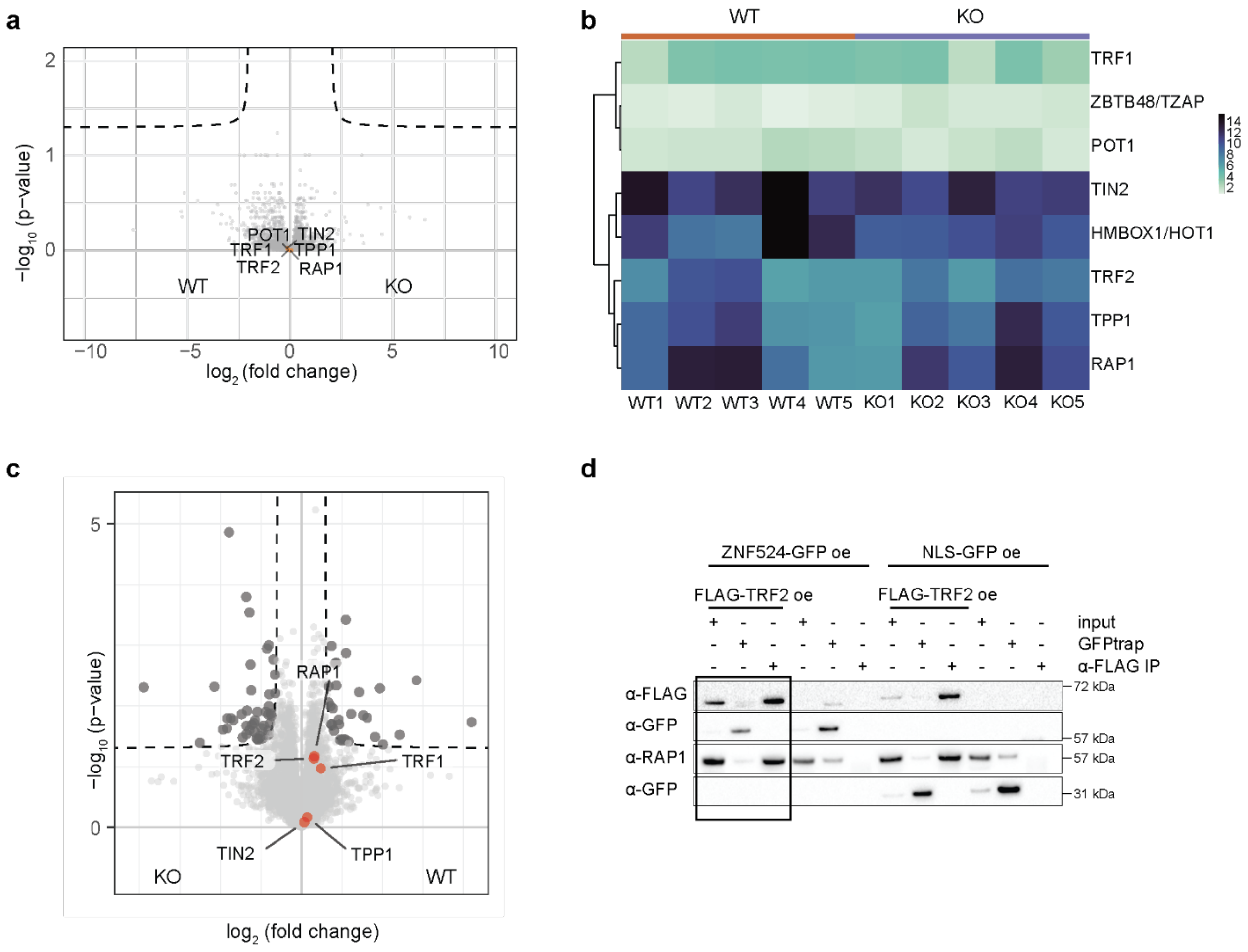


**Supplementary Fig. 7. ZNF524 does not regulate TRF2 and RAP1 expression and does not interact with TRF2**

(**a**) Volcano plot of RNA-seq results in U2OS WT and ZNF524 KO clones. Members of the shelterin complex are highlighted (orange) in the non-differentially regulated genes. 5 WT and 5 KO clones were used (n=5 with each independent clone used as a biological replicate). (**b**) Heatmap of telomere binders and the shelterin complex members identified by RNA-seq in the individual U2OS WT and ZNF524 KO clones. (**c**) Volcano plot of proteome measurements in five U2OS WT and ZNF524 KO clones (n=5 with each independent clone used as a biological replicate). Members of the shelterin complex are highlighted (orange) among the background cloud proteins. (**d**) Co-Immunoprecipitation of ZNF524-GFP and TRF2-FLAG overexpression. IPs and input containing both overexpression constructs are indicated by the black box. NLS-GFP overexpression served as negative control (n=2 with each replicate seeded and transfected independently).

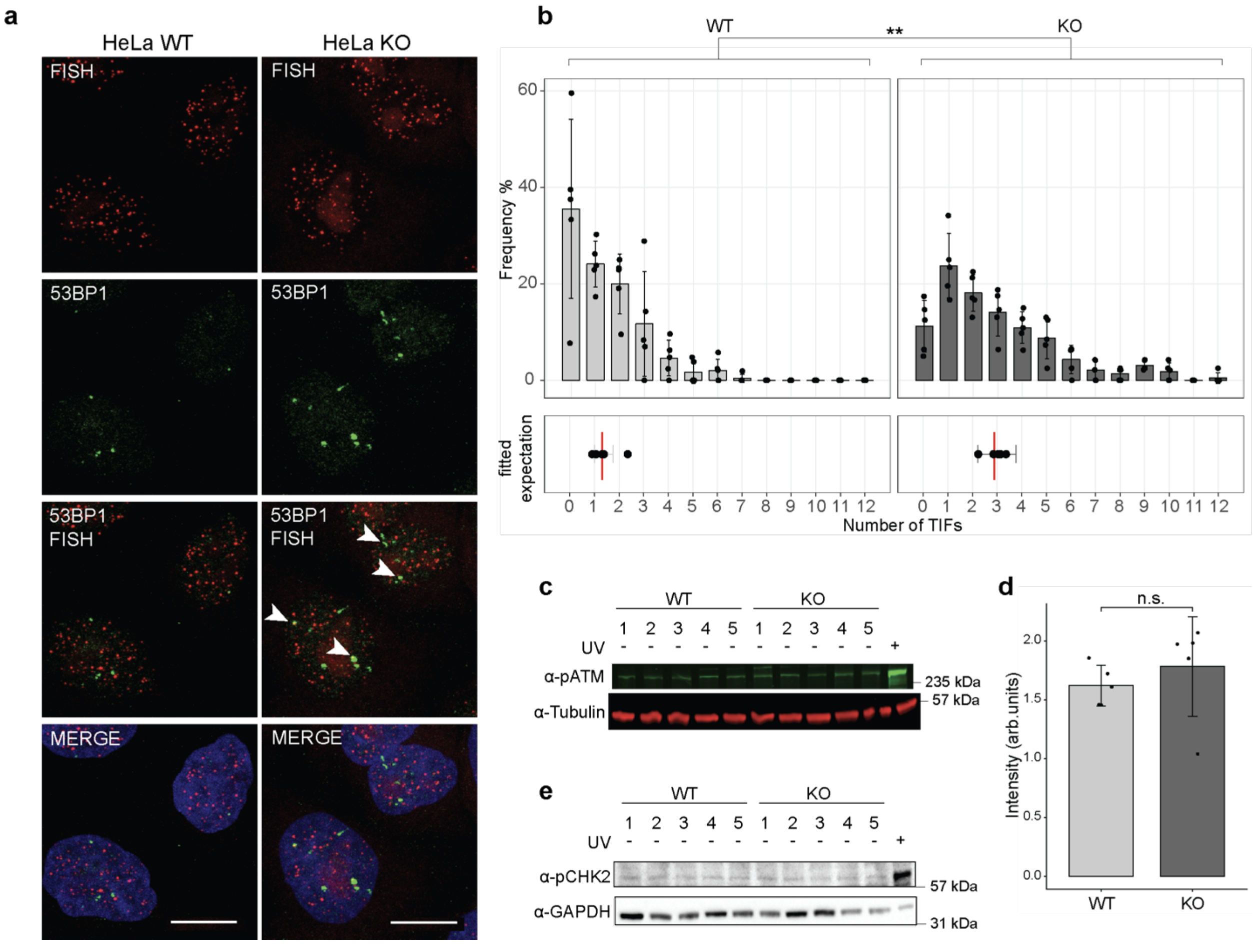


**Supplementary Fig. 8. DNA damage arises upon ZNF524 removal**

(**a**) 53BP1 immunofluorescence staining (green) coupled with telomeric FISH (red) indicates telomere dysfunction induced foci (TIFs, white arrows), scale bars represent 10 µm. Nuclei were counterstained with DAPI (blue). (**b**) Quantification of TIFs per cell in HeLa; 5 WT and 5 KO clones were counted with at least 35 nuclei per clone; upper plot: Frequency of cells with the indicated number of TIFs; error bars represent SD; lower plot: the vertical lines (red) represent the fitted expected number of TIFs (GLMM for negative binomially distributed data). Error bars represent 95% confidence intervals for the mean number of TIFs. The p-value was calculated using a Likelihood Ratio Test; ** $p<0.01$ (n=5). (**c**) Quantitative Western blot showing total pATM protein levels in U2OS WT and ZNF524 KO clones with tubulin as loading control. U2OS cells treated with 40 mJ UV served as positive control. (**d**) Quantification of pATM signal normalized to tubulin. The bar plot shows the mean intensities ± SD. The intensity values of the individual clones are depicted as black dots. Statistical comparison by Welch-test (n=5). (**e**) Western blot

showing total pCHK2 protein levels in U2OS WT and ZNF524 KO clones with GAPDH as loading control. U2OS cells treated with 40 mJ UV served as positive control. For all data, n represents the number of independent clones used as biological replicates.

**Supplementary Table 1. ITC results**

| Protein | $\Delta H$ (kJ per mol) | $-T\Delta S$ (kJ per mol) | N | $K_D$ (μM) |
|---|---|---|---|---|
| ZNF524$^{wt}$ | -64.3 ± 1.10 | 24.7 | 1.03 | 0.09 ± 0.02 |
| ZNF524$^{ZF13}$ | -30.3 ± 1.27 | -4.69 | 1.36 | 0.59 ± 0.15 |
| ZNF524$^{ZF12}$ | -335 ± 0.00 | 315 | 1.21 | >100 |
| ZNF524$^{ZF23}$ | -11.9 ± 1.53 | -21.4 | 1.15 | 1.21 ± 0.79 |
| ZNF524$^{ZF24}$ | -45.7 ± 1.06 | 10.8 | 0.99 | 0.62 ± 0.10 |
| ZNF524$^{ZF34}$ | | | | N.D. |
| ZNF524$^{R131A}$ | -84.2 ± 1.60 | 47.8 | 1.06 | 0.34 ± 0.05 |
| ZNF524$^{R153A}$ | -21.1 ± 0.61 | -17.4 | 0.61 | 0.15 ± 0.04 |
| ZNF524$^{S155A}$ | -46.4 ± 0.68 | 9.34 | 0.90 | 0.25 ± 0.03 |
| ZNF524$^{H156A}$ | -25.4 ± 1.01 | -15.1 | 0.92 | 0.06 ± 0.04 |
| ZNF524$^{R159A}$ | -16.8 ± 1.25 | -22.5 | 0.58 | 0.10 ± 0.09 |
| ZNF524$^{R180A}$ | -41.7 ± 1.08 | 5.86 | 0.75 | 0.41 ± 0.08 |
| ZNF524$^{E181A}$ | -41.8 ± 1.30 | 5.94 | 0.72 | 0.42 ± 0.09 |
| ZNF524$^{E184A}$ | -38.8 ± 1.02 | 1.46 | 0.89 | 0.23 ± 0.13 |
| ZNF524$^{N211G}$ | -65.4 ± 1.04 | 27.3 | 0.93 | 0.17 ± 0.03 |
| ZNF524$^{R215A}$ | -60.0 ± 1.60 | 22.6 | 0.82 | 0.22 ± 0.06 |
| ZNF524$^{ZF2M}$ | | | | N.D. |
| ZNF524 $^{ZF2M(-1-4-7)}$ | | | | N.D |
| ZNF524 $^{ZF2M(-4-5-7)}$ | | | | N.D |
| ZNF524$^{ZF3M}$ | -26.6 ± 0.63 | -11.5 | 0.83 | 0.17 ± 0.04 |
| ZNF524$^{ZF4M}$ | -63.1 ± 1.56 | 24.4 | 0.95 | 0.13 ± 0.04 |

A 12-bp duplex telomeirc DNA (5'-GGTTAGGGTTAG-3') was titrated to ZNF524 ZF1-4 wild type protein or mutations.
ZNF524$^{ZF2M}$: ZF1-4 with R153A/S155A/H156A/R159A mutations;
ZNF524 $^{ZF2M(-1-4-7)}$: ZF1-4 with R153A/H156A/R159A mutations;
ZNF524 $^{ZF2M(-4-5-7)}$: ZF1-4 with R153A/S155A/H156A mutations;
ZNF524$^{ZF3M}$: ZF1-4 with R180A/E181A/E184A mutations;
ZNF524$^{ZF4M}$: ZF1-4 with N211G/R215A mutations.

**Supplementary Table 2. ITC results with telomeric variant repeats**

| Protein | DNA | *ΔH* (kcal per mol) | *-TΔS* (kcal per mol) | N | $K_D$ (μM) |
|---|---|---|---|---|---|
| ZNF524$^{wt}$ | TTAGGG | -15.3 ± 0.26 | 5.9 | 1.03 | 0.09 ± 0.02 |
| ZNF524$^{wt}$ | TCAGGG | -15.0 ± 0.24 | 6.24 | 0.95 | 0.28 ± 0.04 |
| ZNF524$^{wt}$ | TGAGGG | -9.2 ± 0.38 | 0.37 | 0.85 | 0.27 ± 0.11 |
| ZNF524$^{wt}$ | TTGGGG | -9.7 ± 0.46 | 1.14 | 0.81 | 0.33 ± 0.13 |
| ZNF524$^{wt}$ | GTGAGT | | | | N.D. |
| ZNF524$^{wt}$ | $(TTAGGG)_4$-G4 | -14.02 ± 0.28 | 6.59 | 0.28 | 3.58 ± 1.16 |
| ZNF524$^{wt}$ | $(TTAGGG)_4$-ssDNA | | | | N.D. |

12-bp telomeric and telomeric variant DNA duplexes, as well as a negative control 12-bp DNA duplex, were titrated to ZNF524 ZF1-4 wild type protein:
TTAGGG duplex (5'-GGTTAGGGTTAG-3');
TCAGGG duplex (5'-GGTCAGGGTCAG-3');
TGAGGG duplex (5'-GGTGAGGGTGAG-3');
TTGGGG duplex (5'-GGTTGGGGTTGG-3');
GTGAGT duplex (5'-GTGTGAGTGTGA-3').
$(TTAGGG)_4$-G4: 5'-TTAGGGTTAGGGTTAGGGTTAGGG-3' annealed in 20 mM Tris-HCl (pH 7.5), 100 mM KCl and 0.1 mM EDTA to form G4 structure;
$(TTAGGG)_4$-ssDNA: 5'-TTAGGGTTAGGGTTAGGGTTAGGG-3' annealed in 20 mM Tris-HCl (pH 7.5), 100 mM NaCl and 0.1 mM EDTA.

**Supplementary Table 3. Crystallography data collection and refinement statistics**

| | **ZNF524 ZF1-4 in complex telomeric DNA** |
|---|---|
| Wavelength(Å) | 0.978 |
| Space group | $P2_12_12_1$ |
| **Cell parameters** | |
| a, b, c (Å) | 43.81, 52.19, 97.49 |
| α, β, γ (°) | 90, 90, 90 |
| Resolution(Å) | 50.00–2.40(2.44–2.40)[a] |
| $R_{merge}$ (%) | 6.1(37.7) |
| $I/\sigma I$ | 25.5(3.7) |
| Completeness (%) | 99.46 |
| $CC_{1/2}$ | 99.8(90.4) |
| Average redundancy | 6.6(4.7) |
| **Refinement** | |
| No. reflections (overall) | 9162 |
| No. reflections (test set) | 447 |
| $R_{work}/R_{free}$(%) | 21.83/25.33 |
| Number of atoms | |
| Protein | 1545 |
| DNA | 609 |
| $H_2O$ | 90 |
| ZN | 4 |
| B factors (Å$^2$) | |
| Protein | 38.47 |
| DNA | 36.94 |
| $H_2O$ | 33.78 |
| ZN | 33.55 |
| r.m.s. deviations | |
| Bond lengths (Å) | 0.003 |
| Bond angles (°) | 0.599 |
| Rampage plot % residues | |
| Favored | 97.3% |
| Allowed | 2.70% |
| Outliers | 0 |

Data collection and refinement statistics
