# Supplementary material for: ZNF524 directly interacts with telomeric DNA and supports telomere integrity
Source: Nat Commun. 2023 Dec 12;14:8252. doi: 10.1038/s41467-023-43397-7 (PMC10716145; doi:10.1038/s41467-023-43397-7)
Supplement: Supplementary file 3 — Description of Additional Supplementary Files [file 41467_2023_43397_MOESM3_ESM.pdf]

Description of Additional Supplementary Files

File Name: Supplementary Data 1
Description: Proteins identified in BioID assay (ZNF524 vs ZNF524 ZF2 mut)

File Name: Supplementary Data 2
Description: Proteins identified in BioID assay (ZNF524 vs NLS)

File Name: Supplementary Data 3
Description: Genes identified by RNA-seq comparing U2OS WT and ZNF524 KO clones

File Name: Supplementary Data 4
Description: Proteins identified by mass spectrometry comparing U2OS WT and ZNF524 KO clones

File Name: Supplementary Data 5
Description: Primers used in this study

File Name: Supplementary Data 6
Description: Antibodies used in this study
