# Supplementary material for: ZNF524 directly interacts with telomeric DNA and supports telomere integrity
Source: Nat Commun. 2023 Dec 12;14:8252. doi: 10.1038/s41467-023-43397-7 (PMC10716145; doi:10.1038/s41467-023-43397-7)
Supplement: Supplementary file 10 — Reporting Summary [file 41467_2023_43397_MOESM10_ESM.pdf]

nature portfolio

Corresponding author(s): Dennis Kappei, Falk Butter & Fudong Li

Last updated by author(s): Oct 30, 2023

Reporting Summary

Nature Portfolio wishes to improve the reproducibility of the work that we publish. This form provides structure for consistency and transparency in reporting. For further information on Nature Portfolio policies, see our Editorial Policies and the Editorial Policy Checklist.

Statistics

For all statistical analyses, confirm that the following items are present in the figure legend, table legend, main text, or Methods section.

n/a	Confirmed	
☐	☒	The exact sample size (n) for each experimental group/condition, given as a discrete number and unit of measurement
☐	☒	A statement on whether measurements were taken from distinct samples or whether the same sample was measured repeatedly
☐	☒	The statistical test(s) used AND whether they are one- or two-sided<br>*Only common tests should be described solely by name; describe more complex techniques in the Methods section.*
☒	☐	A description of all covariates tested
☒	☐	A description of any assumptions or corrections, such as tests of normality and adjustment for multiple comparisons
☐	☒	A full description of the statistical parameters including central tendency (e.g. means) or other basic estimates (e.g. regression coefficient) AND variation (e.g. standard deviation) or associated estimates of uncertainty (e.g. confidence intervals)
☐	☒	For null hypothesis testing, the test statistic (e.g. F, t, r) with confidence intervals, effect sizes, degrees of freedom and P value noted<br>*Give P values as exact values whenever suitable.*
☒	☐	For Bayesian analysis, information on the choice of priors and Markov chain Monte Carlo settings
☒	☐	For hierarchical and complex designs, identification of the appropriate level for tests and full reporting of outcomes
☒	☐	Estimates of effect sizes (e.g. Cohen's d, Pearson's r), indicating how they were calculated

Our web collection on statistics for biologists contains articles on many of the points above.

Software and code

Policy information about availability of computer code

Data collection	Western blot data: ChemiDoc Touch Imaging System (BioRad).<br>Mass spec data was collected on either a Q Exactive HF (BioID) or Exploris 480 (proteome) using the XCalibur software.
Data analysis	The diffraction data was processed using the HKL2000 software (HKL Research). Experimental phasing was performed with the AutoSol program in the PHENIX suite, with the zinc atom sites being found by SHELX C/D program. The Buccaneer program in the CCP4 suite was utilized for model building, and further manual building and refinements of the protein and the DNA duplex were completed with COOT and Phenix.refine, respectively.<br>Quantitative Western Blots were quantified using Image Studio (LI-COR). Slot blots and IF images were quantified using Fiji (ImageJ). We analyzed the count data of TIF events using a generalized linear mixed model for negative binomially distributed data using the R package lme4. IF images on metaphase chromosome spreads were analysed with the AxioVision software (Zeiss).<br>Mass spec raw files were processed with MaxQuant (for BioID: version 1.5.2.8 & 2.0.1.0; for proteome: version 1.6.5.0).

For manuscripts utilizing custom algorithms or software that are central to the research but not yet described in published literature, software must be made available to editors and reviewers. We strongly encourage code deposition in a community repository (e.g. GitHub). See the Nature Portfolio guidelines for submitting code & software for further information.

Data

Policy information about availability of data

All manuscripts must include a data availability statement. This statement should provide the following information, where applicable:

- Accession codes, unique identifiers, or web links for publicly available datasets
- A description of any restrictions on data availability
- For clinical datasets or third party data, please ensure that the statement adheres to our policy

All data needed to evaluate the conclusions in the paper are present in the paper and/or the Supplementary Materials. The atomic coordinates and structure factors for ZNF524 ZF1-4 in complex telomeric DNA have been deposited in the Protein Data Bank (PDB) under the accession code PDB 7YSF. The mass spectrometry data have been deposited to the ProteomeXchange Consortium via the PRIDE 95 partner repository with the dataset accession codes PXD024630 [https://www.ebi.ac.uk/pride/archive/projects/PXD024630], PXD043084 [https://www.ebi.ac.uk/pride/archive/projects/PXD043084] and PXD031416 [https://www.ebi.ac.uk/pride/archive/projects/PXD031416]. The ChIP-seq data are available under the GEO accession code GSE196661 [https://www.ncbi.nlm.nih.gov/geo/query/acc.cgi?acc=GSE196661]. The RNA-seq data are available in the SRA, BioProject under the accession code PRJNA804430 [https://www.ncbi.nlm.nih.gov/bioproject/?term=PRJNA804430]. Source data are provided with this paper.

Human research participants

Policy information about studies involving human research participants and Sex and Gender in Research.

Reporting on sex and gender	NA
Population characteristics	NA
Recruitment	NA
Ethics oversight	NA

Note that full information on the approval of the study protocol must also be provided in the manuscript.

Field-specific reporting

Please select the one below that is the best fit for your research. If you are not sure, read the appropriate sections before making your selection.

☒ Life sciences ☐ Behavioural & social sciences ☐ Ecological, evolutionary & environmental sciences

For a reference copy of the document with all sections, see nature.com/documents/nr-reporting-summary-flat.pdf

Life sciences study design

All studies must disclose on these points even when the disclosure is negative.

Sample size	No sample-size calculation was performed. The number of independent biological replicates (stated in the figure legends) is at least three fore each experiment to ensure sufficient data for statistical analysis, consistent with the current practise in the field.
Data exclusions	No data were excluded from analysis.
Replication	All experiments were repeated based on independent biological replicates (stated in the figure legend).
Randomization	Due to the nature of the experiments/analyses samples were not randomized.
Blinding	We did not blind operators during the analysis since the experimentators analysed their own data.

Reporting for specific materials, systems and methods

We require information from authors about some types of materials, experimental systems and methods used in many studies. Here, indicate whether each material, system or method listed is relevant to your study. If you are not sure if a list item applies to your research, read the appropriate section before selecting a response.

Materials & experimental systems

n/a	Involved in the study
☐	☒ Antibodies
☐	☒ Eukaryotic cell lines
☒	☐ Palaeontology and archaeology
☒	☐ Animals and other organisms
☒	☐ Clinical data
☒	☐ Dual use research of concern

Methods

n/a	Involved in the study
☐	☒ ChIP-seq
☒	☐ Flow cytometry
☒	☐ MRI-based neuroimaging

Antibodies

Antibodies used

All antibodies used in this study are listed in Supplementary Data 6 including catalog numbers and dilutions for specific applications.
The following primary antibodies were used:
GFP; Mouse; 11814460001; Merck; WB 1:1,000; IF 1:2000-5000
FLAG; Rabbit; F7425; Sigma-Aldrich; WB: 1:800
FLAG; Mouse; F3165; Sigma-Aldrich; IP: 2.5ug
TRF2; Mouse; NB100-56506; Novus; WB: 1:1,000; IF: 1:250
TRF2; Rabbit; NB110-57130; Novus; WB: 1:1,000; IF: 1:250
RAP1; Mouse; ab14404; Abcam; IF: 1:250
53BP1; Rabbit; NB100-304; Novus; IF: 1:250-500
TRF1; Mouse; PCRP-TERF1-1E5; DSHB; IF:1:50
POT1; Rabbit; NB500-176; Novus; IF: 1:100
Tubulin; Mouse; E7-s; DSHB; WB: 1:200
Actin; Rabbit; A2066; Sigma-Aldrich; WB: 1:500
GAPDH; Mouse; 2G7; DSHB; WB: 1:200
ZNF524; Rabbit; selfmade; WB: 1:300
pATM; Rabbit; ab81292; Abcam; WB: 1:5,000
pCHK2; Rabbit; 2661T; Cell signaling technology; WB: 1:2,000

The following secondary antibodies were used:
Rabbit; IgG; Donkey; NA934V; GE Healthcare; WB: 1:3,000; Horse-radish peroxidase
Mouse; IgG; Sheep; NA931V; GE Healthcare; WB: 1:3,000; Horse-radish peroxidase
Rabbit; IgG; Goat; 926-32211; LI-COR; WB: 1:15,000; IRDye® 800CW
Mouse; IgG; Goat; 926-68070; LI-COR; WB: 1:15,000; IRDye® 680RD
Mouse; IgG; Donkey; A31571; Thermo Fisher Scientific; IF: 1:800; AlexaFluor647
Rabbit; IgG; Donkey; A31573; Thermo Fisher Scientific; IF: 1:800; AlexaFluor647
Mouse; IgG; Goat; A11017; Thermo Fisher Scientific; IF: 1:5,000; AlexaFluor488 (F(ab)2)
Rabbit; IgG; Goat; A21246; Thermo Fisher Scientific; IF: 1:5,000; AlexaFluor647 (F(ab)2)
Mouse; IgG; Goat; A-11032; Thermo Fisher Scientific; IF: 1:500; AlexaFluor594
Rabbit; IgG; Goat; A-21206; Thermo Fisher Scientific; IF: 1:300; AlexaFluor488
Mouse; IgG; Donkey; A21202; Thermo Fisher Scientific; IF: 1:1,000; AlexaFluor488
Rabbit; IgG; Donkey; A21207; Thermo Fisher Scientific; IF: 1:1,000; AlexaFluor594

Validation

The self-made ZNF524 antibody was validated for specificity by comparing ZNF524 WT and KO cell lines (Suppl. Fig. 3). The remaining antibodies are commercially available and are documented on the manufacturers' websites including prior use and validation in other publications:

GFP; 11814460001; Merck; manufacturer note: cited in >900 publications
FLAG; F7425; Sigma-Aldrich; manufacturer note: cited in >2,700 publications
FLAG; F3165; Sigma-Aldrich; manufacturer note: cited in >7,800 publications
TRF2; NB100-56506; Novus; manufacturer note: Publications confirming reagent in 5 species (Human, Mouse, Rat, Marsupial, Muntjac) and in 8 applications: (Chemotaxis, ICC, ICC/IF, IF, IHC-P, IP, PLA, WB)
TRF2; NB110-57130; Novus; manufacturer note: Publications confirming reagent in 3 species (Human, Mouse, Rat) and in 10 applications (Chemotaxis, Cytometric Bead Assay Standard, ELISA, FISH, ICC/IF, IF/IHC, IP, Knockdown, PLA, WB)
RAP1; ab14404; Abcam; manufacturer note: Product tested in IP, WB and Flow Cytometry applications
53BP1; NB100-304; Novus; manufacturer note: Publications confirming reagent in 11 species)Human, Mouse, Rat, Avian, Drosophila melanogaster, Goat, Naked mole-rat, Porcine, Primate, Rabbit, Reptile) and in 18 applications (ChIP, Chemotaxis, FISH, FLOW, IB, ICC/IF, IF, IF-FISH, IF/IHC, IHC, IHC-Fr, IHC-P, IP, ISH, KD, KO, PLA, WB)
TRF1; PCRP-TERF1-1E5; DSHB; confirmed telomeric pattern in our IF experiments
POT1; NB500-176; Novus; manufacturer note: Publications confirming reagent in 1 species (Human) and in 2 applications (Chemotaxis, WB)
Tubulin; E7-s; DSHB; manufacturer note: cited in >100 publications
Actin; A2066; Sigma-Aldrich; manufacturer note: cited in >3,000 publications
GAPDH; 2G7; DSHB; manufacturer note: validated in 5 publications for WB
pATM; ab81292; Abcam; manufacturer note: Product tested in IP, WB, IHC and Flow Cytometry applications
pCHK2; 2661T; Cell signaling technology; manufacturer note: cited in >600 publications

Eukaryotic cell lines

Policy information about cell lines and Sex and Gender in Research

Cell line source(s)	U2OS, HeLa (Kyoto), GM847, Saos2 and WI-38 VA-13 are from our lab internal stocks and had been originally authenticated. HeLa 1.3 and HT1080ST were earlier gifts from the de Lange lab (based on publication PMID 9476899) and Lingner lab (based on publication PMID 16424902), respectively, as stated in the Methods in section.
Authentication	The parental U2OS line and individual WT and ZNF524 KO clones were authenticated by STR profiling at the German Collection of Microorganisms and Cell Cultures (DSMZ). The remaining cell lines were authenticated by STR profiling by !st BASE.
Mycoplasma contamination	Cell lines used in this study were regularly tested fto be negative for mycoplasma.
Commonly misidentified lines (See ICLAC register)	No commonly misidentified cell lines were used in this study.

ChIP-seq

Data deposition

☒ Confirm that both raw and final processed data have been deposited in a public database such as GEO.

☒ Confirm that you have deposited or provided access to graph files (e.g. BED files) for the called peaks.

Data access links<br>*May remain private before publication.*	The ChIP-Seq data is available under the GEO identifier GSE196661.<br>https://www.ncbi.nlm.nih.gov/geo/query/acc.cgi?acc=GSE196661<br>Access token: mxufkoscxduprmh
Files in database submission	GSM5897806 Input DNA - ZNF524-GFP WT<br>GSM5897807 Input DNA - ZNF524-GFP ZF2 mut<br>GSM5897808 Input DNA - NLS-GFP<br>GSM5897809 ChIP ZNF524-GFP WT rep 1<br>GSM5897810 ChIP ZNF524-GFP WT rep 2<br>GSM5897811 ChIP ZNF524-GFP WT rep 3<br>GSM5897812 ChIP ZNF524-GFP ZF2 mut rep 1<br>GSM5897813 ChIP ZNF524-GFP ZF2 mut rep 2<br>GSM5897814 ChIP ZNF524-GFP ZF2 mut rep 3<br>GSM5897815 ChIP NLS-GFP rep 1<br>GSM5897816 ChIP NLS-GFP rep 2<br>GSM5897817 ChIP NLS-GFP rep 3
Genome browser session (e.g. UCSC)	As described in the manuscript the ZNF524 ChIP-seq data did not yield any peaks beyond the described enrichment of telomeric reads (which are counted rather than conventional peaks due to their repetitive nature). Therefore, we do not provide a genome browser session.

Methodology

Replicates	For each experimental condition (ZNF524-GFP WT, ZF2 mutant & NLS) 3 replicates were sequenced along with 1 input sample for each condition.
Sequencing depth	For each sample 39 to 52 million reads were obtained as 150bp paired-end reads.
Antibodies	GFP-Trap magnetic agarose beads (Chromotek; gtma)
Peak calling parameters	ChIP-seq reads were aligned to human reference genome version GRCh38 using Bowtie2 version 2.3.5.1 with default settings<br>Reads having a mapping quality less than 40 were filtered out using samtools version 1.12<br>Peaks were called using MACS version 2.2.7.1 in paired-end mode with default q-value cut-off (0.05) and the respective inputs were used as control.<br>bigwig files were generated using deeptools version 3.5.0, it was normalized using CPM with binsize 6, smoothlength 30 and reads centered option.
Data quality	Quality check of the fastq files was done using FASTQC. 0-2 peaks were detected across all samples using MACS.
Software	Custom code was used to run the above stated command line tools. Most of the tools were installed with the help of Conda.
